# "We’re Not Providing the Best Care If We Are Not on the Cutting Edge of Research": A Research Impact Evaluation at a Regional Australian Hospital and Health Service

**DOI:** 10.34172/ijhpm.2022.6529

**Published:** 2022-05-22

**Authors:** Amy Brown, Alexandra Edelman, Tilley Pain, Sarah Larkins, Gillian Harvey

**Affiliations:** ^1^Townsville Hospital and Health Service, Townsville, QLD, Australia.; ^2^James Cook University, Townsville, QLD, Australia.; ^3^Flinders University, Adelaide, SA, Australia.

**Keywords:** Research Investment, Research Impact, Research Capacity, Regional Hospital, Realist-Informed Evaluation, Australia

## Abstract

**Background:** Research is central to high functioning health services alongside clinical care and health professional training. The impact of embedded research includes delivery of high-quality care and improved patient outcomes. Evaluations of research impact help health service leadership ensure investments lead to the greatest healthcare benefits for patients. This study aimed to retrospectively evaluate the impact of research investment from 2008 to 2018 at Townsville Hospital and Health Service (THHS), a regional Hospital and Health Service (HHS) in Queensland, Australia. The evaluation also sought to identify contextual conditions that enable or hinder intended impacts.

**Methods:** A mixed-methods realist-informed evaluation was conducted using documentation, interviews with 15 staff and available databases to identify and measure research investments, impacts and contextual conditions influencing impact outcomes.

**Results:** Between 2008 and 2018, THHS increased resources for research by funding research projects, employing research personnel, building research-enabling facilities, hosting research events, and providing research education and training. Clinical practice, policy and workforce impacts were successful in isolated pockets, championed by individual researchers and facilitated by their policy and community-of-practice networks. However, there was little organisational-level support for continuity of research and implementation into practice and policy. Availability of research supports varied geographically across THHS, and across disciplines.

**Conclusion:** Definitive steps in the development of THHS as a credible and productive research centre and leading hospital research centre in Northern Australia are evident. Continuing investments should address support for the research continuum through to translation and establish ongoing, systematic processes for evaluating research investment and impact.

## Background

**Key Messages**

** Implications for policy makers**

- The findings of this study underscore the importance of systematic data collection to enable tracking of research investments and research activity, research capacity, clinical practice and policy, health workforce and patient/population health impacts from research.
- To achieve and sustain clinical practice, workforce and health impacts from research, research support structures within healthcare organisations should include systematised support for research through to translation across the organisation in addition to support for short-term research projects.
- Other regional healthcare organisations might adopt a similar realist-informed evaluation approach as used in this study to enable identification of contextual enablers and barriers specific to their unique health service characteristics.

** Implications for the public**

Translating research findings into practice can drive improvements in healthcare delivery. We evaluated research investment, activity and impact at a regional Hospital and Health Service (HHS) within Australia. There was evidence of increased research activity, but there were variations across the organisation and changes in health service practice resulting from research were not systematically supported. Data to enable assessment of impact were also very limited. Implications for policy, practice and research include three bodies of work that should be taken forward in the Townsville Hospital and Health Service (THHS) to achieve its research development and impact goals, including patient care and health impacts from research: (1) developing a systematic data collection framework, (2) further increasing research support and (3) supporting research translation. The findings of the study, along with the impact evaluation approach taken, are likely to be useful for other healthcare organisations interested in examining research investment and impact.

Alongside clinical care and health professional education, research is a core function of health services in providing quality healthcare. A wide range of benefits are associated with embedding research in healthcare, including delivery of better quality of care and improved patient outcomes.^1,2^ Evaluations of the impact of research are essential to help health service leadership (ie, executive and senior managers) ensure that investments lead to the greatest impacts in healthcare for patients.^3-5^

 In Australia, research capacity, capabilities and funding within healthcare organisations have been traditionally clustered within large metropolitan health services, with less research-related capacity and resources in regional, rural and remote settings.^3,6^ Several policy initiatives to build health research capacity in rural areas have occurred over the recent decade.^7^ Some of these initiatives have reported great success, but geographic inequities in health research capacity remain, meaning people living outside of major cities miss out on the health and health system benefits attributable to embedded research.^8,9^ Efforts to continue to increase research capacity and capability in regional Hospital and Health Service (HHS) settings are essential if regional institutions are to participate in and benefit from growing national efforts to embed research into health service delivery.^10,11^

 Despite the growing interest and public investment in embedding research capacity within healthcare organisations in Australia, few impact evaluations in healthcare settings are reported. In addition, very few published studies offer insights into research capacity development within healthcare settings outside of metropolitan settings in Australia.^12^ This paper contributes to addressing these gaps by reporting on a research impact evaluation undertaken at the Townsville Hospital and Health Service (THHS) located in northern Queensland, Australia. The aim of the study was to retrospectively evaluate research investment and impact within THHS from 2008 to 2018 by addressing the following research questions:

1. What are the key features and milestones of the research investment and development journey at THHS?
2. What are the goals of THHS’s research investment?
3. What impacts have resulted from the research investment?
4. What contextual factors have enabled or hindered these impacts?
5. How do the identified impact pathways link to theories of research impact and knowledge mobilisation within healthcare settings?

###  Study Setting and Context

 THHS is a statutory authority delivering healthcare services to a population of approximately 250 000 people across an area of around 150 000 square kilometres.^13^ The population served by THHS is highly diverse, and includes populations living in the large regional city of Townsville, which is home to the tertiary referral Townsville University Hospital, and populations living in regional, remote and very remote locations with access to smaller hospital facilities and visiting services. THHS works collaboratively with other providers to deliver primary, secondary, and tertiary care services to patients in the region, with a total of 775 beds in 2018. The annual budget of THHS was AU$960 million, and the organisation employed 6248 staff in 2018.^13^

 The research development journey at THHS has involved a gradual, organic approach to developing research capacity with research becoming a key strategic pillar of the health service alongside clinical care and education.^14^ THHS’ research ambition is “to be the leading hospital research centre in Northern Australia, translating novel research into innovative, high-quality patient care” (THHS Research Strategy 2018-2022; p. 1). Further information about the study setting, and a description of the research development journey at THHS, is published in an earlier paper.^14^

## Methods

###  Study Design

 A mixed methods study design was used consisting of three phases, with data collection undertaken from February 2019 to February 2020. The study aimed to identify contextual influences on impact pathways and drew from realist evaluation methods to identify what works, for whom and in what circumstances.^15^ This involved the development of context-mechanism-outcome (CMO) configurations to explain the relationships between investments in research at THHS and various types of impact.The study adopts a broad interpretation of “research” incorporating all studies requiring ethical review, implementation focussed research, quality improvement and clinical audits.

###  Data Collection

 Data collection was undertaken in two separate phases. Phase 1 of the study addressed research questions 1 and 2 by describing the research trajectory at THHS, identifying goals relating to research, and developing a program theory and conceptual framework to guide Phases 2 and 3 (outlined below). The methods and findings from Phase 1 are reported in an earlier paper and include the conceptual framework and research impact evaluation structure that guided Phase 2.^14^ Phase 1 involved interviews with six current and former health service executives and senior employees, and organisational document review.

 Phase 2 of the study involved collecting quantitative data from routine data sources at THHS and qualitative data from documentation and interviews to identify impact examples, trends and contextual conditions. Quantitative data were accessed from a range of THHS sources, which were selected with reference to the Phase 1 evaluation structure. These sources were pre-existing repositories of data routinely collected and reported by THHS staff for research-related administration and reporting. The Townsville Research, Education, Support and Administration (TRESA) Unit within THHS provided most of this data including several spreadsheets relating to: research ethics and governance applications and approvals; projects awarded and funded through the Study, Education Research Trust Account (SERTA); research training initiatives; and personnel occupancy reports. Data were extracted from the three published THHS Research Annual Reports (2015/2016; 2017; 2018) on infrastructure, publications, events, grants, and research project vignettes, and the THHS Pharmacy Department provided data on pharmacological clinical trials.

 Phase 2 interviewees were purposively selected to achieve maximum variation by THHS Service Group (functional work units within THHS with separate governance and roles, see footnote of Table 1), professional discipline, research experience level and gender (Table 1). An expansive list of research-active staff and managers was compiled from which an initial list of 15 potential interviewees was selected. These individuals were contacted by email by a member of the research team to gauge interest, and 10 agreed to be interviewed. A snowballing method was used to identify a further five individuals who were approached and agreed to be interviewed during the data collection period. Interviews were semi-structured and supported by an interview guide (Supplementary file 1) informed by the framework developed in Phase 1 of the study. One member of the research team (AE) conducted the interviews which were undertaken in person apart from one phone interview. All interviews were recorded and transcribed verbatim, except one which was recorded by the interviewing researcher using handwritten notes at the request of the interviewee. All interviewees provided written consent.

**Table 1:** Characteristics of Interviewees in the Study (Phase 2)

**Characteristic**		**Number of Interviewees** **(N = 15)**
THHS Service Group^a^	Health and wellbeing	2
	Medical	6
	Mental health	1
	Rural	1
	Surgical	3
	Non-clinical	2
Professional discipline	Allied health	3
	Nursing	4
	Medicine	6
	Managerial	1
	Research support	1
Research experience level	Novice	5
	Emerging	2
	Established	7
	Non-researcher	1
Gender	Female	8
	Male	7

^a^Service Groups (SG) overview: *Health and Wellbeing SG* includes gynaecological, maternity, newborn and children’s services, primary care in community, prisoner health, aged care, and oral health. *Medical SG* includes specialities and services such as emergency medicine, internal medicine, neurology, infection diseases, endocrinology, repository, gastroenterology, pharmacy and comprehensive cancer services. *Mental Health SG* provides comprehensive mental health assessment and treatment services including acute inpatient care, crisis intervention and community integration and health rehabilitation, forensic mental health, and case management. *Rural Health SG* provides public health access (such as emergency, general medicine and general surgery) to the populations of surrounding smaller towns including Ingham, Charters Towers, Ayr and Home Hill. *Surgical SG* provides perioperative services including surgery, anaesthetics, and theatre care, with diagnostic care including medical imaging. (Source: Townsville HHS Annual Report 2017/2018).

 Phase 3 of the study was conducted concurrently with the other phases and involved verification of the study findings with key stakeholders including target end users of the research in THHS. Verification processes undertaken throughout the project included meetings with senior staff, member checking of all interview transcripts, and regular project updates to the THHS executive. Summaries of draft Phase 2 findings were also shared with the interviewees involved in Phase 1 and feedback was sought. The draft Phase 2 findings were presented to clinician researchers at THHS at a research event and audience members were provided with an opportunity to ask questions and contact the research team with any further questions or feedback. The intent of these verification processes was to ensure the target end users of the study findings had the opportunity to comment on and reflect on emerging findings at every stage of the study, reflecting best practice co-production methods.^16,17^

###  Data Analysis

 Interview transcripts and notes from Phase 2 were read and re-read by three researchers (AB, AE and TP). Inductive coding, involving the development of concepts to stand for interpreted meaning of the data,^18^ was undertaken by one researcher (AE) using NVivo [QSR Version 12]. These codes were then grouped under deductive themes corresponding to the broad fields of the interview guide. Reflecting the guide and Phase 1 framework, both qualitative and quantitative data were used to identify examples and trends across the six categories identified in Phase 1 of: research investments; research activity impacts; research capacity impacts; clinical practice and policy impacts; health workforce impacts; and patient and population health impacts.

 Interview data were also used to identify contextual influences acting to enable or hinder impacts from research investment. Inductive themes relating to contextual enablers and barriers were discussed and refined during regular meetings of the research team. Initially, thirteen inductive themes were developed.

**Figure 1:**
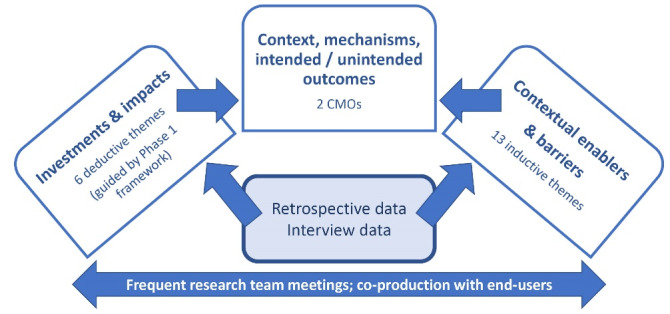
Analysis Process Used to Identify Investments, Impacts and CMOs. Abbreviation: CMOs, context-mechanism-outcomes.

 Both the deductive and inductive themes were then consolidated into two CMO configurations incorporating both intended and unintended outcomes. Figure 1 shows the analysis process used to identify investments and impacts and develop the CMOs.

 Finally, feedback received from stakeholders in Phase 3 was collated into a single report which was used to inform the final analysis and write up of results.

###  Reflexivity

 The research team brought a wide range of health disciplines (medicine, nursing, allied health, and health policy) and professional experiences to the project. The team has varying levels of research experience, with two researchers being international experts in qualitative research methods with realist evaluation experience. Three members of the team were employees at THHS at the time of the study. To reduce the influence of pre-existing knowledge on the interpretation of findings, the researchers used purposive sampling to select interviewees, the interview guide was used in the same way for all interviews and transcripts and notes were read and re-read by several members of the research team. Codes and emerging findings were discussed in detail among all team members. In addition, the inductive approach to theme development relating to contextual enablers and barriers allowed the participants’ voices to direct this part of the analysis. Care was taken during interactions with stakeholders that the findings remained grounded in the data, with feedback from verification processes in Phase 3 augmenting the researchers’ understanding of the findings within the THHS context.

## Results

**Figure 2:**
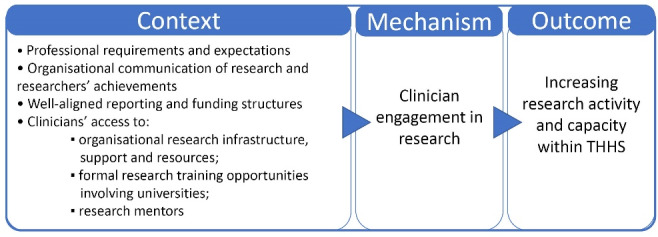
CMO1 – Clinician Engagement. Abbreviations: CMO, context-mechanism-outcome; THHS, Townsville Hospital and Health Service.

**Figure 3:**
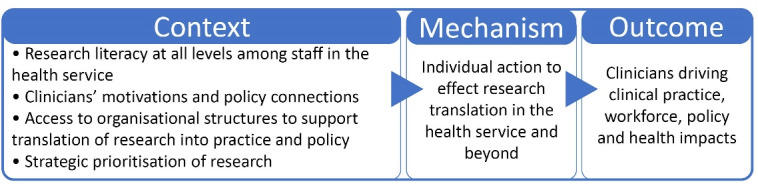
CMO2 – Action to Effect Research Translation. Abbreviation: CMO, context-mechanism-outcome.

 Results are presented against the two CMOs identified in the study (Figures 2 and 3) and are supported by quantitative trends and interview quotes.

###  CMO1 – Clinician Engagement in Research 

**Figure 4:**
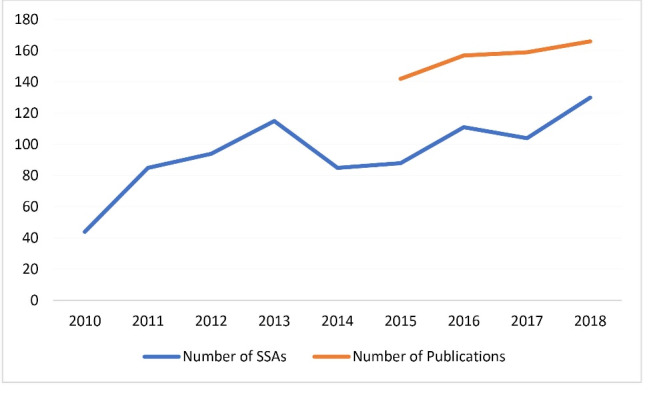
Number of site-specific approvals (SSAs) at THHS, 2010-2018; and Publications by THHS Clinician Researchers, 2015-2018. Abbreviations: SSAs, site-specific approvals; THHS, Townsville Hospital and Health Service. Note: Systematic publication data was not available prior to 2015. Data source: TRESA 2019, THHS Research Annual Reports 2015/2016; 2017; 2018.

 Indicators of research investment and activity show an increase in activity between 2008 and 2018. The number of site-specific approvals (SSAs) for research in THHS tripled from 44 approved studies in 2010 to 130 in 2018. Publications by THHS clinician researchers (at all levels from novice to established) also increased, though at a slower rate, from 142 in 2015 to 166 in 2018 (Figure 4). These increases are likely to reflect greater clinician engagement in research over time.

 Clinician engagement in research required several contextual elements at THHS. These elements were additive, meaning the presence of one or two contextual elements enabled enactment of the mechanism and additional elements further increased the likelihood of clinician engagement in research.

 A key contextual enabler of clinician engagement in research was the availability of research infrastructure, support and resources for research which increased between 2008 and 2018 at THHS. Specifically, there was increased funding and support for research projects, research personnel, research-enabling facilities, research events and research education and training. For example, more than $3.3 million in SERTA funds was distributed by THHS to research projects and capacity-building between 2013 and 2018, just over 70 percent of which supported applied clinical research projects including clinical trials (TRESA 2019). SERTA grants enabled some clinician researchers to access backfill (suitably trained staff) for their substantive clinical roles so they could conduct research projects, and the grants also served to legitimise their research time.

 “*[SERTA funding] didn’t always mean that it [a clinical position] got backfilled, but I suppose that funding there was for them [managers] to see. It gave me that dedicated time as I was learning – I’d done a Grad Cert in Research at that point” *(Int 10).

 Clinicians’ access to research support was also boosted by the establishment of TRESA in 2015 and employment of several administrative and research fellow staff. For example, increased assistance and support with administrative elements of research such as ethics and governance requirements and submissions were considered helpful for researchers. In parallel, the Aboriginal and Torres Strait Islander Health Leadership Advisory Committee, encouraged to review research proposals by TRESA, was described as an initiative that strengthened Aboriginal and Torres Strait Islander voices in research and research processes (Int 8).

 “*So people are engaging in it [research] more, and also because they do know there is a support network, there’s a research office upstairs, there’s research fellows, there’s people that have done research before” *(Int 1).

 “*I think the other thing that scares them [new researchers] a lot is the paperwork and I think the ethics approval process scares a lot of people off. I think having someone that is familiar with that process and can help them navigate that, rather than have them just sent a link and saying, ‘fill it out’ [has helped]” *(Int 5).

 Access to formal research training opportunities had also increased over time. TRESA began providing research training in 2016 as a lunch-time series over 12 weeks, or a two-day block mode. This research training was described as a useful enabler of research engagement (Int 9), covering aspects such as legislative frameworks, research design and ethics and governance processes. THHS staff attendance at this training increased from 25 in 2016 to 80 in 2018, including some virtual attendance from Queensland Health staff external to THHS.

 The Cohort Doctoral Studies Program at neighbouring James Cook University (JCU) was mentioned by several interviewees in relation to accessing formal research degrees. This Program provides higher degree research students with a scheduled program of workshops and a peer-support network. It appealed to THHS clinicians because it made balancing clinical and family commitments with research more feasible, encouraging them to embark on formal research training.

 “*The thought came across about doing the PhD and the only reason I actually enrolled in the PhD was because of the Cohort Program at JCU. So that enabled me to work full-time […] I think it was the link – having that support – so you know this was six years ago, there was still that stigma around when you do a PhD you’re on your own” *(Int 1).

 Novice and emerging researchers also described becoming interested in research for the first time after working with more senior clinicians and academics at THHS and collaborating universities, who were sometimes described as mentors. This exposure led clinicians to consider pursuing research including through formal research training.

 “*There was a senior fellow there [at THHS] at the time, who has now moved onto another hospital, and she was very supportive of me looking beyond the quality improvement cycle and starting to try and think about asking those [research] questions” *(Int 10).

 “*So I started talking to people with an interest and an expertise over at JCU and about three people in meetings, three relatively senior people, said ‘wow, that’s really interesting, there’s a PhD in that’. They all said that. And I thought, wow you know that sounds really big, and I thought well I’ll do the research, whether or not it translates into a PhD is another matter […] and I’ve flirted with the idea of doing a PhD and haven’t given up on that” *(Int 4).

 At an undergraduate level, the introduction of research components into health professional courses served to expose younger cohorts of graduates to research. In turn, they were seen to be more likely to understand the “value” of research than older cohorts who did not receive this early exposure (Int 8). In areas of THHS where research was actively encouraged and achievements celebrated, staff felt more confident to engage in research.

 “*If there is an explicit expectation or encouragement in the role for research, and there is the capacity for them to spend some of their time doing research, that is a huge barrier that is then overcome. Because they feel like they can do it” *(Int 13).

 Professional drivers external to THHS and universities also encouraged some clinicians to become involved in research. For some staff, a key driver was the inclusion of research in employment awards, contracts and college training programs as a professional development requirement. For others, involvement in research was driven by a more general professional expectation that clinicians should keep on top of the literature and evidence in their fields. Use of research as a differentiator in professional fields with large numbers of graduates was a motivator for some.

 “*[Some staff] are building it [research] into their role, and certainly in position descriptions, there’s the named requirement to contribute to research and to improve the body of knowledge” *(Int 9).

 “*The other thing that has driven research and post-graduate qualification is the employment competition […]. So anything that you’ve got that is gonna stand out on your CV, is a bonus” *(Int 1).

 These external supports and incentives encouraged some individuals in THHS to develop strong research track records, with several becoming recognised as national and international leaders in their fields. Over time, research communities of practice had formed in some departments/disciplines, with research increasingly becoming an expected part of clinicians’ roles across the HHS.

 “*I certainly think it [research] is a lot more visible than it has been in the past. […] there is a focus on it now, and it is the topic of discussion now more so than ever” *(Int 7).

 “*I think that more people are actually doing research, and it feels like, particularly outside of the medical areas, like for our Service Group, I think it’s picked up” *(Int 9).

 “*[Research at THHS] has changed over the years, and I think dramatically over the past five years after a lot of improvement in terms of ethics, and also the funding and support from the research unit as well, which has gone remarkably very well […] It is making Townsville to be seen as a leader in north Queensland, I mean in northern Australia in particular, in terms of research” *(Int 2).

 Some of the contextual enablers, however, were manifest as barriers if they were absent, or implemented unevenly across the HHS, or not fit for purpose. Several interviewees felt more investment and support was needed in THHS for staff to be able to replace their clinical time with research time and described differences in the capacity to commit to research between clinical streams. Some interviewees reflected that executive and senior managers may be unaware of how much personal time, over and above allocated professional development time, was required for research. Several interviewees reflected that professional development time was very limited, with little time for research and precedence given to clinical commitments.

 “*I don’t think you’d survive in this [research] area without that personal sacrifice and dedication. And some of us are much better at knowing the limits of that, but that’s a choice, and you do wonder if they [executive staff] acknowledge that” *(Int 10).

 “*I think it was like 16 hours [allowance] through your whole year to do a project […] Sometimes it was honoured, sometimes it wasn’t […] all of a sudden something would change and you were there on the unit [doing clinical work]” *(Int 10).

 More protected time for research was seen by some to be needed for clinicians with no prior research experience, and for those in smaller work units.

 “*I think [what is needed is] probably protected time to immerse yourself in getting an understanding of it for a start […]” *(Int 15).

 Some interviewees also felt more support was needed for externally led projects in the form of project management and grant support to help with research design, project administration and access to funding.

 “*If you have dedicated project officers, then their job is really to convert ideas into research questions, and then with projects, so then the trainees come with projects already levelled up; or if senior clinicians have ideas, when the trainees come and ask for a project, you’ll then bring this person in and say, ok convert to a research question and then go on with it” *(Int 11).

 “*One of the big things that we’ve been asking for years is for a research manager […] to help do the paperwork and all those other things […]” *(Int 6).

 While research engagement was increasing overall across THHS, there was some variability in access to support between disciplines, service groups, work units and service locations, resulting in different levels of research activity and capacity. Staff in some work units, and notably those located in rural services outside of Townsville, reported less research infrastructure and capacity than others.

 “*You need to make people aware that [the] Townsville research centre […] belongs to everywhere, not just the hospital staff […] we are isolated, but we are all part of that unit and [we need] some way to link those things” *(Int 12).

 “*They [the Townsville-based research enabling staff] are not coming down, they’re not in our face and encouraging us, [although] we know that they’re there […] We’ve [previously] had various people come down and try to encourage us and involve us in research, and we were quite interested but just with the nature of our work and the busyness, and I think lack of knowledge, we never really got going. Like we had good ideas for research projects but factoring it into our work it’s, been pretty much impossible” *(Int 15).

 The way research supports were provided was important in influencing accessibility. For example, access to funding for clinical backfill was not helpful if no one could be recruited to the role because of workforce shortages (which are particularly acute in rural areas) or difficulties in recruiting staff to regional areas.

 “*It’s all well and good [when] someone is able to access a grant that supports the service to backfill from a financial perspective, it’s then being able to find the appropriately skilled person to fill it […] it’s becoming increasingly difficult to recruit […]” *(Int 7).

 An overarching contextual barrier to research engagement was that research was not directly incentivised through the THHS funding model or within Service Agreements. Accordingly, the challenge for managers was to balance supporting research with meeting service commitments, which often involved a trade-off.

 “*The service requirements of the institution are always there and present, and sometimes the managers are supportive of research in word but not in kind […] the organisation says ‘we want research’, but I don’t know that it’s gonna put the cheque book down and say ‘well have some money to fund it’, that doesn’t happen” *(Int 4).

 “*I think a lot of the clinical services are now running on the smell of an oily rag. And it’s a case of, if we can’t release someone for a clinical secondment to somewhere else, it’s gonna be very difficult as a line manager to justify release of someone to be able to go and do something that doesn’t fulfil a clinical gap somewhere else” *(Int 7).

 In parallel, some researchers felt that research was often secondary to clinical work. Many described not having enough time to do research or to engage in research-related events, or doing research in their own time (Int 6). In all, the reporting and funding structures led to research being a lower priority than clinical and administrative functions across THHS, which was a barrier to research engagement.

 “*It’s very hard to manage that [clinical and administrative responsibilities] and have lots of time for these research ideas. So that’s perhaps one barrier to why I haven’t gotten as far as what I thought […] Usual business is activity – usual business is the nitty gritty of getting patients in, getting them out, getting the care provided” *(Int 4).

 “*[For] many people, there’s so much clinical work, that frankly there’s no way they’re going to be able to do any research. So there has to be a bit of encouragement while understanding that service is clearly key” *(Int 14).

 Overall, the contextual enablers of research engagement over time in THHS likely contributed to increasing research activity as indicated by increasing publication numbers and SSAs in THHS. Further increases to research activity are therefore possible if the barriers to research engagement are addressed.

###  CMO2. Action to Effect Research Translation

 In addition to enabling clinician research engagement, translating research into clinical practice and policy change was a key strategic aspiration of THHS. Overall, interviewees strongly felt research was essential for THHS to be a high functioning health service and to improve clinical care and patient outcomes.

 “*[Research is] critical in ensuring that all of the care we provide within THHS is evidence-based, […] we’re not providing care, or the best care, or genuinely keeping patients safe if we are not on the cutting edge of research” *(Int 8).

 “*Good healthcare needs to be evidence based, and the only way we can have quality evidence is for health professionals to critically engage with, and that means doing, but also being informed by, quality research. So at the very least, clinical people need to have the ability to appraise research, so not just believe it, but appraise it, so have the skills to be able to do that […] that’s crucial for the health of the population” *(Int 13).

 Several contextual elements enabled or hindered individuals in THHS to undertake actions to translate research into healthcare and other impacts. Like the contextual elements in CMO 1, these were additive, and were also manifest as barriers if they were absent or not fit-for-purpose. A key enabler of research translation was attainment, among staff at all levels, of a base level of understanding about what research is and the processes needed for research impact. Research literacy among THHS executives and senior management was particularly important because it underpinned organisational support for research in the face of tensions (described earlier) between THHS research aspirations and misaligned reporting/funding models and requirements.

 “*Because a lot of the people in those positions [executive and management roles] haven’t done research, they don’t know the output from it […] if they need to lift their service, the first thing to go is research usually […] I think it’s absolutely crucial they understand the basic methodology of how a question is formulated, how data is collected and what the outcomes do, and how that translates back into practice” *(Int 1).

 A few executive and director-level staff were described by interviewees as drivers of the growth of THHS research activity over the years. Furthermore, it was these individuals who championed research and made many of the key investment decisions. THHS leadership were seen to have become more supportive of research over time, with some commencing research training themselves. As a result, research, as a strategic pillar of the health service, was becoming more widely accepted as a legitimate way for clinicians to spend their time.

 However, there was a strong sense among interviewees that executive staff and board members sometimes had unrealistic expectations about research, which could hamper ongoing research engagement of staff or organisational investment in translation pathways.

 “*I do wonder if there’s a mismatch between their expectation – to what [a] $20 000 [SERTA grant] will actually get you. […] So perhaps - maybe they won’t sustain that funding the next time you come back and apply for it [SERTA funds], if they don’t see that you’ve met their expectations the previous time” *(Int 10).

 Research literacy at a mid-management level was also important because managers made decisions about resource commitments within their own departments. However, there were differences in the value placed on research in different departments and work units. For example, one rural location reported less support from management.

 “*Some of the [management], for example in rural and remote areas, they couldn’t understand clearly that this [research] is meant to improve their care. […] So we have had this as a challenge which is drawing us back in terms of conducting research in rural and remote areas” *(Int 2).

 Understanding research helped clinicians see the value of it in the workplace, but the level of research literacy across THHS was seen to vary considerably.

 “*I think there’s a lack of understanding of what it [research] is and how it can help to improve us […] And I think part of that is showing to the people who do the work, how to translate the research to practice” *(Int 15).

 In addition to the variability of research literacy which influenced research impact expectations, there were few structures and incentives to support systematic research translation research within THHS. Despite increasing support in THHS for clinician engagement in research (see CMO 1), comparatively little attention was given to supporting and incentivising research translation into clinical, policy and workforce impacts at an organisational level. A key challenge for researchers engaging in translation was the relatively short-term support for research projects, with little ongoing encouragement or tangible support for continuity of research and implementation into practice. It was acknowledged by some that translation requires a range of additional effort, investment, and skills.

 “*We have a commitment to rolling out the best practice related to research […] I think it’s probably one of the hardest things to do, is to actually make research live into new practice, or change practice to match research. I think you have to – it is a change management process really. So you need to treat it like a change management project” *(Int 9).

 “*The expectation is that we just do it [implement the findings of research into practice…but] as soon as it comes to say where I need an extra dollar to provide this service, it’s ‘oh well no money for that […]’” *(Int 4).

 Some interviewees observed a lack of organisation-wide enabling structures or research capacity at the crucial “mid-level” to enable translation to occur.

 “*So you have that [THHS vision] statement […] and then all these disconnects […] So there is complete disconnect in through the middle levels […] there is all these enablers and barriers – [more] needs to be driven through the middle levels” *(Int 11).

 “*We do have a high-level research committee in the hospital, and that’s sort of almost at an executive level, below that we don’t really have much […]. So it is very scattered at the low level, there’s not much at the mid-level, but there is a higher-level research committee” *(Int 1).

 Structures to facilitate translation need clear articulation of risks and accountabilities relating to implementation of a clinical practice change. However, several interviewees reflected the culture at THHS was often risk-averse, and this had negative consequences for both research engagement and translation. Risk aversion was apparent in both staff attitudes and organisational policies and procedures. Busy clinical work schedules compounded this cultural reluctance to try something new in practice.

 “*A reason probably why it [clinical practice change] doesn’t happen so much is that people in this hospital are really afraid about accountability […]. You’ve got a great project and you can change practice, if we do this, and if something happens, who’s accountable? And a lot of people can’t answer that question, and when you can’t answer it, nothing happens” *(Int 1).

 “*Some people care about it [translating research into practice] a little, enough to be involved and supportive, but most of us just want to get on with our job […], we’re expected to do more and more with less and less, and at some point, you just can’t get blood out of a stone” *(Int 4).

 Despite limited translation enablers, individual clinicians’ recognition and concern for health and service challenges motivated some to embark on research with a direct bearing on their practice. Some interviewees described key intrinsic motivators as: seeing disadvantage in the community (Int 4); and noticing administrative or clinical processes that could be delivered better in their clinical environment (Int 3; Int 6). Indeed, the desire to improve services for patients was described as an underlying motivation for all research efforts at THHS (Int 3).

 Several researchers, particularly at senior levels, were driving clinical and policy change from their research by leveraging their individual policy networks such as involvement on boards and committees that included policy-makers and end users of their research. In many cases, these connections resulted in translation of research findings into clinical guidelines and other policy documents. Table 2 summarises some examples of actions taken by individual researchers at THHS to translate research in a variety of ways, including co-production, choice of research topic/question arising from clinical need, governance infrastructure, collaboration and policy and professional linkages, as identified by interviewees and in research annual reports.

**Table 2:** Translational Actions Undertaken in THHS Research Projects That Were Reported to Deliver Clinical, Workforce and/or Policy Impacts at THHS

**Area of Translational Action**	**Description and Examples**
Choice of research topic	Pursuing research that responds directly to an observed population health or health service problem. Examples: High rates of diabetes-related amputations among rural, remote and Indigenous populations in the north Queensland region led to research in THHS on implementation of innovative equipment in wound care. THHS did not have a policy in skin injuries for neonates, prompting research on epidemiology of skin injuries from mechanical force in neonates and the development of a specific policy and risk assessment tool.
Co-production	End-users included in all phases of the research from research design. Examples: Following a quality assurance audit on pain referrals among Aboriginal and Torres Strait Islander clients at THHS, a consumer engagement strategy was developed involving active collaboration between THHS staff, the Townsville Aboriginal and Islander Health Service and the broader community to identify barriers to pain clinic referrals and hospital access among Indigenous community members.
Governance infrastructure	Establishment of governance infrastructure, including memoranda of understanding, networks and centres, to formalise and coordinate multi-stakeholder relationships and funding. Examples: The Australasian Teletrial Model, developed by the Clinical Oncology Society of Australia, is operationalised through a collaboration between the Townsville Cancer Centre, Queensland Cancer Clinical Network and the Health, Innovation, Investment and Research Office of the Department of Health, building from THHS-led research.
Multi-disciplinary collaboration	Collaboration between clinicians from multiple different disciplines working across departments: THHS clinicians established monthly multi-disciplinary teleconference meetings in 2018 between hospital surgeons, and diabetes, endocrinology and orthotics specialists from THHS and North West HHS to discuss prevention, treatment and management of limb amputations in the region drawing from current research.
Communication	Ongoing communication of findings through multiple mediums targeting academic, clinical, policy and broader public stakeholders. Examples: Inclusion of published and presented findings on medical management for aortic aneurysm in the European Society of Cardiology Guidelines on the Diagnosis and Treatment of Aortic Diseases (2014). Findings from a tele-health service enhancement model implemented within THHS actively communicated to other departments in THHS, published in peer-reviewed journals and presented at conferences, resulting in establishment of the Queensland Remote Chemotherapy Solution Model.
Policy and professional linkage	Ongoing policy and community of practice engagement at local, state, national and international levels. Examples: Co-authorship of the CDC-sponsored guideline (2013) on Diagnostics for melioidosis and the CDC-sponsored guidelines for treatment and post-exposure prophylaxis of this condition (2010). Active leadership and involvement in key state, national and international groups and organisations such as Health Services Research Association of Australia and New Zealand Executive, and the Australasian Tele-trial Consortium and Queensland Tele-Trial Working Group.

Abbreviations: THHS, Townsville Hospital and Health Service; HHS, Hospital and Health Service; CDC, Centres for Disease Control.

 However, translational actions tended to be driven by individuals for their own research or professional networks and not by an overarching organisational strategy that articulated or incentivised priority areas of research with impact potential in THHS.

 “*That’s been my focus, for a range of reasons that really are around the fact that it’s both clinically and research-wise a big, neglected space […] I’m very mindful of those gaps. Those clinical gaps. And I’ve been made aware of the research gaps, subsequently [… there is] no organisational strategy, I think it’s more so personal interest” *(Int 4).

 Accordingly, clinical, workforce and policy impacts from research at THHS were identifiable mostly in small, isolated pockets of change promoted by the clinician leading the research, rather than being enabled as part of usual practice across the health service.

## Discussion

 The organisational, service quality and health benefits of research within healthcare organisations are widely recognised in the literature.^1,2,19,20^ This empirical research impact evaluation at the THHS responds to growing efforts to embed research within health services in Australia, including in regional, rural and remote areas. While the research development journey at THHS is still underway and commenced later than in many urban organisations, this study identifies substantial progress between 2008 and 2018 in developing research activity and capacity, with world-leading research achievements in areas linked to its regional, rural and remote location and catchment population. Pain et alsimilarly found increased research activity in allied health clinicians at THHS over four years.^21^ The current study also identified several examples of clinical practice, policy, workforce and health impacts from THHS clinicians’ research. In addition, several contextual elements were identified in the study that are enabling or hindering the achievement of research impact goals at THHS. This study refined the contextual elements identified in Phase 1 of the study (see Supplementary file 2).^14^

 Two CMO configurations were developed which describe relationships between research investment at THHS and impacts from research. Several contextual elements were identified that influence clinicians’ research engagement and enactment of research translation at THHS, with resultant increases in research activity and capacity, as well as pockets of clinical, workforce, policy and health impacts. While some contextual elements enabled research impacts to occur, the same elements were also barriers to research impact if they were absent, unevenly implemented or not fit for purpose, giving rise to unintended outcomes such as variations in research and translation activity across professional streams, service groups and sites within the organisation.

 Despite research activity at THHS increasing overall, the study identified variation across professions and sites, with gaps most evident in rural health services in relation to access to supports for clinician research engagement. While an overarching need for more, and different, types of research support were identified, the findings indicate that existing investments could be more targeted, building from an understanding of local gaps and needs and leveraging the growing evidence in the literature on supporting research engagement in clinical settings. For example, practicalities such as limited workforce “backfill” for clinical roles were found to limit research capacity in rural areas and supports were less accessible in the rural and remote THHS locations. Evidence from other studies shows that implementing research fellow positions, allocating resources and offering access to research education, mentoring and networking improves research engagement among rurally-based clinicians.^22–24^ As such, the current study identifies scope within THHS to adapt, test and scale up these and other research engagement strategies to address unmet needs.

 In addition, despite being key research impact aspirations at THHS,^14^ clinical, workforce, policy and health impacts from research are visible only in isolated pockets of success, championed by individual researchers and facilitated by their own policy and community-of-practice networks. A key finding of this study, therefore, is that organisation-wide, systematic processes to enable research translation have not yet been established, and several factors inhibit translational pathways. In general, research support at THHS tends to be for relatively short-term research projects, with little ongoing support for continuity of research and implementation into practice and policy. These findings are not wholly unexpected or unique by national or international standards given the widely recognised challenges involved in systematising research translation.^25-27^ For this reason, testing, evaluation and scale-up of effective and sustainable systems to enable systematic research translation at THHS would position it at the forefront of such efforts nationally and internationally. Investing in translation and research capacity building is also likely to be critical to position THHS to access major new funding schemes for research within Australia’s health system, noting the focus in the new Medical Research Future Fund Strategy (2021-2026) on translation to deliver impact in areas of unmet need and to improve health system efficiency and effectiveness.^11^

 Translation of research in clinical settings requires preconditions for change in clinical practice and is dependent on the ability to manage and shape contexts, requiring human agency.^28^ Accordingly, transitioning a health service with pockets of translation and stretched resources to one in which translation is systematised at every level is likely to be challenging, particularly in a dispersed regional, rural and remote healthcare context. The current study identified areas of translational action from impactful projects (choice of research topic aligned with health need and service priority; co-production with end-users; governance infrastructure supporting multistakeholder collaboration; multi-disciplinary collaboration; communication mechanisms; and policy and professional linkage) that align with other published work,^29^ demonstrating that innovative approaches developed elsewhere could be trialed with success in THHS.

 The “engaged scholarship approach” of seeking partnerships between academics and clinical knowledge users in addressing health system needs is one approach that is being tested globally.^30,31^ THHS, through its involvement in the Tropical Australian Academic Health Centre (TAAHC), is well-placed to lead best practice approaches to improving research translation using such collaborative governance models. At the time of the study, TAAHC was still developing its strategy and approach to facilitating research and translation across the northern Queensland region, and as such was still a peripheral consideration for study participants at best. Going forwards, TAAHC is likely to be a valuable vehicle through which THHS clinicians and collaborating university academics might build national and international networks and develop multi-disciplinary proposals to access funding for local research and impact programs.^10^ Further studies applying existing implementation and behaviour change could be undertaken by THHS for specific research and translation programs, and for ongoing impact measurement.

 Evaluation is critical to ensure that investments in research lead to an intended impact. There are growing calls to measure and monitor research effectiveness, efficiency and equity within healthcare and health systems.^33,34^ Within THHS, there are increasing efforts to measure research activity against new metrics in addition to “traditional” research grants and publications metrics, but there are important gaps. For example, there are no routine, organisational measures of research capacity building in THHS, despite the well-recognised importance of building research capacity within clinical settings, both within THHS and in the literature.^35^ There are also no processes in THHS to enable systematic data collection on clinical practice, policy, workforce and health impacts from research. These gaps could be addressed with reference to the burgeoning literature on research impact evaluation, which include both linear “pipeline” models of research translation and associated impact metrics as well as more nuanced (but resource-intensive) approaches that explore causal pathways, as adopted in the current study.^36^ Addressing these gaps in routine data collection needs to be a priority for THHS going forward to enable ongoing assessment of progress towards its research impact goals.

###  Implications for Policy, Practice and Research

 There are several implications of this research for policy, practice and research. Within THHS, the findings highlight a need for a systematic research impact data collection framework to support routine tracking of a range of research impact types into the future. Reflecting the research impact evaluation structure developed in Phase 1 of the study,^14^ such a framework should incorporate both quantitative and qualitative indicators of research investment, research activity impacts, research capacity impacts, clinical practice and policy impacts, health workforce impacts and patient health impacts. Examples of research activity, capacity and clinical practice impact indicators that are likely to be useful in the future include: volume, citations and characteristics of THHS publications; volume and characteristics of external research grants received by THHS staff; involvement of staff in journal review and editorships; and use of THHS-led research in clinical guidelines and health professional education material. Several impact evaluation frameworks in the literature, such as the Canadian Academy of Health Sciences Impact Framework,^37^ offer detailed lists of indicators that could be used by THHS to guide such future data collection. Noting the difficulties faced by some clinicians in justifying time spent on research away from clinical care, and the absence of research key performance indicators in the THHS Service Agreement, future data collection in THHS should also consider ways to routinely examine health service efficiencies attributable to research. As noted in Phase 1, there are many impact frameworks in the literature, with the best approaches likely combining linear and more complex realist evaluation components.^14^ There is an opportunity to take this body of work forward in collaboration with other health services in northern Queensland (including through TAAHC).

 Second, the findings relating to barriers to research engagement suggest a need to identify and address issues inhibiting research participation among specific disciplines or groups of THHS staff. Strategies might include establishment of clear clinician-academic career pathways across all clinical streams, and clear articulation within work units of expectations relating to balancing research with clinical work. Targeted support for rurally based clinicians to undertake research is particularly needed to ensure that remote, chronic, community and rehabilitative models of care used in THHS incorporate mechanisms to improve practice through research. Research on models of care and service delivery outside of the hospital setting, noting the broader context in which THHS operates and substantial unmet population health needs across the northern Queensland region, is likely to require a different set of tools, skills and approaches to hospital-focused research.^10^

 Third, there is a need for support structures to be established in THHS to systematise research translation across the organisation, while building current research support. Support for research and dissemination beyond the initial project phase would facilitate communication of findings to a range of target audiences including policy stakeholders, as would actively supporting clinicians to establish and maintain linkages with communities of practice and policy fora. Creation of one or more translation pathways within THHS might also help in identification and discussion of options to change clinical practice considering emerging research evidence. Finally, priority research themes, requiring articulation of impact pathways, might help to incentivise research efforts that demonstrate impact potential.

###  Strengths and Limitations

 This study contributes to the knowledge base on embedding research and optimising research impact within health service settings, offering a unique perspective from a regional tertiary health service in northern Queensland. By enacting co-production processes with target end-users, the researchers hope that findings will contribute to the ongoing development of THHS into one of regional Australia’s foremost research-infused teaching health service entities. In addition, few studies examining research impact within healthcare settings draw on realist evaluation methods or focus on health services outside of major cities. The evaluation framework developed in Phase 1 of this project also has potential utility for other regional healthcare organisations.^14^

 Limitations include the inability of the project to identify and examine research investment and impact trends for all impact types of interest, as this was hampered by a lack of systematic organisation-wide data collection systems. Despite some relatively new and valuable data sources available against some research activity impact indicators, very limited data were available for: human resources investments; some research capacity impacts; and clinical, policy, workforce, and health impacts. In addition, as the purposive interviewee selection approach adopted in the study aimed to elicit a broad picture of key issues across the organisation, future research might seek to investigate in greater depth the specific research engagement gaps and barriers facing particular groups of clinicians within THHS. This would help in identification and trialing of targeted investment options aimed at achieving various types of impact.

## Conclusion

 While the goal of becoming the leading hospital research centre in northern Australia remains aspirational, definitive early steps in the development of THHS as a credible and productive research centre are evident. Investments in research-enabling infrastructure at THHS are valued highly by staff but need to be supplemented by further practical support, particularly in rural areas, and clear clinician-researcher career pathways. Continuing investments should also involve actively supporting research translation and establishing ongoing, systematic processes for evaluating research investment and impact. Without these additional efforts, aspirations about the promise of *“cutting edge of research” *as a driver of “best care” are unlikely to be fully realised in THHS.

## Acknowledgements

 The authors thank the interviewees involved in the project for their time and thoughtful insights. The authors also acknowledge and thank the staff in the Townsville Research Education Support and Administration Unit, who took the time to identify and forward available data to the research team to support this project.

## Ethical issues

 Ethics approval was granted by Prince Charles Hospital Human Research Ethics Committee (Project ID 44195). All interview participants provided signed informed consent.

## Competing interests

 AB and TP are current employees of the THHS. AE was an employee of the THHS at the time of the study. SL and GH declare that they have no competing interests.

## Authors’ contributions

 AE conceived and designed the study with input from AB and TP. AE and AB wrote the first draft of the manuscript. AE undertook data collection, coding and analysis, with input from AB and TP. All authors reviewed and provided critical feedback at each stage of design, analysis and manuscript development and approved the final version of the manuscript.

## Funding

 This work was supported by the THHS through an annual competitive research grant programme (the Study, Education and Research Trust Account Grant Scheme). All stages of the study were undertaken independently by the researchers.

## Supplementary files


Supplementary file 1. Interview Guide.

Supplementary file 2. Refinements to Contextual Influences Following Phase 2 Data Analysis.

## References

[1] Boaz A, Hanney S, Jones T, Soper B (2015). Does the engagement of clinicians and organisations in research improve healthcare performance: a three-stage review. BMJ Open.

[2] Ozdemir BA, Karthikesalingam A, Sinha S (2015). Research activity and the association with mortality. PLoS One.

[3] Deeming S, Searles A, Reeves P, Nilsson M (2017). Measuring research impact in Australia’s medical research institutes: a scoping literature review of the objectives for and an assessment of the capabilities of research impact assessment frameworks. Health Res Policy Syst.

[4] Buykx P, Humphreys J, Wakerman J (2012). ‘Making evidence count’: a framework to monitor the impact of health services research. Aust J Rural Health.

[5] Hanney S, Greenhalgh T, Blatch-Jones A, Glover M, Raftery J (2017). The impact on healthcare, policy and practice from 36 multi-project research programmes: findings from two reviews. Health Res Policy Syst.

[6] Australian Government Department of Health. Review to Strengthen Independent Medical Research Institutes. Australian Government Department of Health; 2015.

[7] Perkins DA, Barclay L, Browne KM (2011). The Australian Rural Health Research Collaboration: building collaborative population health research in rural and remote NSW. N S W Public Health Bull.

[8] Barclay L, Phillips A, Lyle D (2018). Rural and remote health research: does the investment match the need?. Aust J Rural Health.

[9] Greville H, Haynes E, Kagie R, Thompson SC (2019). ‘It shouldn’t be this hard’: exploring the challenges of rural health research. Int J Environ Res Public Health.

[10] McKeon S. Strategic Review of Health and Medical Research in Australia. Australian Government, Department of Health and Aging; 2013.

[11] Australian Medical Research Advisory Board. Australian Medical Research and Innovation Strategy 2021-2026 Determination. Australian Government, Department of Health and Aging; 2021.

[12] Edelman A, Taylor J, Ovseiko PV, Topp SM (2019). “’Academic’ is a dirty word”: intended impact pathways of an emerging academic health centre in tropical regional Australia. Int J Health Plann Manage.

[13] Queensland Government. Townsville Hospital and Health Service Annual Report. 2018. https://www.townsville.health.qld.gov.au/. Accessed February 22, 2021.

[14] Edelman A, Brown A, Pain T, Larkins S, Harvey G (2020). Evaluating research investment and impact at a regional Australian Hospital and Health Service: a programme theory and conceptual framework. Health Res Policy Syst.

[15] Pawson R, Tilley N. Realistic Evaluation. SAGE Publications Ltd; 1997.

[16] Nguyen T, Graham ID, Mrklas KJ (2020). How does integrated knowledge translation (IKT) compare to other collaborative research approaches to generating and translating knowledge? Learning from experts in the field. Health Res Policy Syst.

[17] Williamson A, Tait H, El Jardali F (2019). How are evidence generation partnerships between researchers and policy-makers enacted in practice? A qualitative interview study. Health Res Policy Syst.

[18] Charmaz K. Constructing Grounded Theory: A Practical Guide through Qualitative Analysis. SAGE Publications; 2006.

[19] Harding K, Lynch L, Porter J, Taylor NF (2017). Organisational benefits of a strong research culture in a health service: a systematic review. Aust Health Rev.

[20] Hanney S, Boaz A, Jones T, Soper B (2013). Engagement in research: an innovative three-stage review of the benefits for health-care performance. Health Services and Delivery Research.

[21] Pain T, Petersen M, Fernando M (2018). Building allied health research capacity at a regional Australian hospital: a follow-up study. Internet J Allied Health Sci Pract.

[22] Pain T, Plummer D, Pighills A, Harvey D (2015). Comparison of research experience and support needs of rural versus regional allied health professionals. Aust J Rural Health.

[23] Webster E, Thomas M, Ong N, Cutler L (2011). Rural research capacity building program: capacity building outcomes. Aust J Prim Health.

[24] O’Sullivan B, Cairns A, Gurney T (2020). Exploring how to sustain ‘place-based’ rural health academic research for informing rural health systems: a qualitative investigation. Health Res Policy Syst.

[25] Braithwaite J, Boyling C, Warwick M, Smith KL, Zurynski Y, Hibbert P. Where the Rubber Meets the Road: A Learning Health Care System for Australia: a Working Paper Providing a Synthesis of Ideas Drawn from Selected Literature Where the Rubber Meets the Road: A Learning Health Care System for Australia. CHRIS WORKING PAPER #106. Australian Institute of Health Innovation; 2019.

[26] Morain SR, Kass NE, Grossmann C (2017). What allows a health care system to become a learning health care system: results from interviews with health system leaders. Learn Health Syst.

[27] Wolfenden L, Yoong SL, Williams CM (2017). Embedding researchers in health service organizations improves research translation and health service performance: the Australian Hunter New England Population Health example. J Clin Epidemiol.

[28] Myall M, May C, Richardson A (2021). Creating pre-conditions for change in clinical practice: the influence of interactions between multiple contexts and human agency. J Health Organ Manag.

[29] Oliver K, Innvar S, Lorenc T, Woodman J, Thomas J (2014). A systematic review of barriers to and facilitators of the use of evidence by policymakers. BMC Health Serv Res.

[30] Barratt H, Shaw J, Simpson L, Bhatia S, Fulop N (2017). Health services research: building capacity to meet the needs of the health care system. J Health Serv Res Policy.

[31] Bowen S, Botting I, Graham ID (2019). Experience of health leadership in partnering with university-based researchers in Canada - a call to “re-imagine” research. Int J Health Policy Manag.

[32] Michie S, van Stralen MM, West R (2011). The behaviour change wheel: a new method for characterising and designing behaviour change interventions. Implement Sci.

[33] Hinrichs-Krapels S, Grant J (2016). Exploring the effectiveness, efficiency and equity (3e’s) of research and research impact assessment. Palgrave Commun.

[34] Rubio DM (2013). Common metrics to assess the efficiency of clinical research. Eval Health Prof.

[35] Cooke J (2005). A framework to evaluate research capacity building in health care. BMC Fam Pract.

[36] Greenhalgh T, Raftery J, Hanney S, Glover M (2016). Research impact: a narrative review. BMC Med.

[37] Panel on Return on Investment in Health Research. Making an Impact: A Preferred Framework and Indicators to Measure Returns on Investment in Health Research. Canadian Academy of Health Sciences; 2009.

