# Supplementary material for: "We’re Not Providing the Best Care If We Are Not on the Cutting Edge of Research": A Research Impact Evaluation at a Regional Australian Hospital and Health Service
Source: Int J Health Policy Manag. 2022 May 22;11(12):3000–11. doi: 10.34172/ijhpm.2022.6529 (PMC10105178; doi:10.34172/ijhpm.2022.6529)
Supplement: Supplementary file 1 — Interview Guide. [file ijhpm-11-3000-s001.pdf]

Article title: "We're Not Providing the Best Care If We Are Not on the Cutting Edge of Research": A Research Impact Evaluation at a Regional Australian Hospital and Health Service

Journal name: International Journal of Health Policy and Management (IJHPM)

Authors' information: Amy Brown[1]¶*, Alexandra Edelman[1,2]¶ , Tilley Pain[1,2] , Sarah Larkins[2], Gillian Harvey[3]

[1]Townsville Hospital and Health Service, Townsville, QLD, Australia.

[2]James Cook University, Townsville, QLD, Australia.

[3]Flinders University, Adelaide, SA, Australia.

(*Corresponding author: Email: amy.brown@health.qld.gov.au)

¶Both authors contributed equally to this paper

Supplementary file 1. Interview Guide

Abbreviations:
ABF: Activity Based Funding; HDR: Higher Degree Research; JCU: James Cook University; QH: Queensland Health; THHS: Townsville Hospital and Health Service

Impact type/contextual factor	Issue to explore	Possible questions
	Introduction	What is your current/former roles at THHS?
Impact - Research activity	Types of research/ quantity	What sort of research do you do?<br>How many research projects have you been involved in over 10 years?<br>(How many student projects (including Honours and PhD or Masters projects) have occurred within your department over 10 years?<br>How many research projects have occurred within your department over 10 years?<br>What types of research are being done in your area?)
Impact - Research capacity	How access to research supports influence career choices and progression<br>Training – benefits and challenges, implications for career progression	What supports are available to you to develop a research career?<br>Has support at THHS helped you access other (external) sources of support? How? Examples?<br>What research training options are available to you?<br>What else is necessary in addition to education and training to become a clinician researcher? Is THHS providing this support?
Impact - Clinical practice change	Support for clinical/policy impact<br>How research is integrated into day-to-day practice<br>Examples of successes/failures	**Can you give a specific example where a research project has led to a change in clinical practice or service provision?**<br>**What did this change look like?**<br>**Can you describe how you think this change came about?**<br>**How long has this change in practice been sustained?**<br>**Were there any factors that enabled this change to occur? What were they?**<br>**Were there any factors that hindered the change? What were they?**<br>**Was this experience shared with others? How?**<br>**How has THHS as an organisation facilitated this type of research to this point?**<br>**What would you like to see more/less of?**<br>**What do you think could improve this process next time?**
Impact - Clinical workforce change	Workforce more clinically capable<br>Greater recruitment	Have you noticed any changes to your practice capabilities because of your research capability?<br>(Do you notice any changes in your colleagues/staff capabilities as a result of their research involvement?)<br>Has research activity helped with recruitment and retention of clinicians? Can you give examples?
Impact - Improved patient and population health	Reported health outcome impacts	Can you give a specific example where a research project has led to patient/population health improvements?<br>How was this measured/assessed?
Contextual factor - Interests, motivations and attitudes	Drivers of research aspiration/engagement (or lack thereof)	What drove your interest in research? / Are you interested in being involved in research?<br>What are your main motivations for engaging in research?

Abbreviations:
ABF: Activity Based Funding; HDR: Higher Degree Research; JCU: James Cook University; QH: Queensland Health; THHS: Townsville Hospital and Health Service

Impact type/contextual factor	Issue to explore	Possible questions
	What motivates/doesn’t motivate<br>Learning requirements	How do you see yourself achieving your research aspirations?
Contextual factor - Visible signals of research value and valued research types	How research is promoted<br>How research is monitored/evaluated<br>How clinicians react to this	How is research promoted in THHS?<br>How is research evaluated/monitored in THHS?<br>How does THHS show it values research?<br>Do these signals have an impact on your current engagement with research? (or on your thoughts about engaging or not engaging in research in the future?) How?
Contextual factor - Characteristics of clinical role	Barriers/enablers to being involved in research relating to clinical role	What are the main enablers and barriers to engaging in research?<br>How do you balance clinical/research work?<br>Are there any requirements for you to be involved in research (e.g. industrial agreements, colleges)? How are these requirements met?<br>(How do you help your staff to balance clinical/research work?)
Contextual factor – research culture and inter-disciplinary collaboration	What sort of culture/way of working promotes implementation of research to practice	Is there a particular culture/way of working that facilitates clinical/policy impacts from research?
Contextual factor – Leaders’ understanding of research	How the leadership at THHS facilitates/inhibits clinical impacts	Does the leadership at THHS understand research?<br>What effect does this have on research and research implementation/translation at the health service?
Contextual factor - Responsiveness to patient and population health concerns	How research prioritised<br>How research fits with strategic vision of THHS	How did you choose your research field/topics?<br>How does your research fit with colleagues’ research and with broader program of research at THHS?<br>How does your research map with patient/population health needs and priorities?<br>What role did THHS have in driving your research focus?<br>What do you think continued THHS support for research activity can or will achieve in the future?<br>What do you think the goals of this support and investment should be?
Contextual factor - Reporting and funding structures	How ABF model and reporting requirements to QH affect the research effort at THHS	How do you balance health system performance drivers with research aspirations and requirements?<br>How does this influence your support for research?

Abbreviations:
ABF: Activity Based Funding; HDR: Higher Degree Research; JCU: James Cook University; QH: Queensland Health; THHS: Townsville Hospital and Health Service
