# Supplementary material for: "We’re Not Providing the Best Care If We Are Not on the Cutting Edge of Research": A Research Impact Evaluation at a Regional Australian Hospital and Health Service
Source: Int J Health Policy Manag. 2022 May 22;11(12):3000–11. doi: 10.34172/ijhpm.2022.6529 (PMC10105178; doi:10.34172/ijhpm.2022.6529)
Supplement: Supplementary file 2 — Refinements to Contextual Influences Following Phase 2 Data Analysis. [file ijhpm-11-3000-s002.pdf]

10.34172/ijhpm.2022.6529

Article title: "We're Not Providing the Best Care If We Are Not on the Cutting Edge of Research": A Research Impact Evaluation at a Regional Australian Hospital and Health Service

Journal name: International Journal of Health Policy and Management (IJHPM)

Authors' information: Amy Brown[1]¶*, Alexandra Edelman[1,2]¶ , Tilley Pain[1,2] , Sarah Larkins[2], Gillian Harvey[3]

[1]Townsville Hospital and Health Service, Townsville, QLD, Australia.

[2]James Cook University, Townsville, QLD, Australia.

[3]Flinders University, Adelaide, SA, Australia.

(*Corresponding author: Email: amy.brown@health.qld.gov.au)

¶Both authors contributed equally to this paper

Supplementary file 2. Refinements to Contextual Influences Following Phase 2 Data Analysis

10.34172/ijhpm.2022.6529

Contextual influences identified in Phase 1	Related contextual influences identified in Phase 2	Description of refinements
Interests, motivations and attitudes of clinicians	Exposure to research mentors<br><br>Access to research training opportunities involving universities<br><br>Access to research support infrastructure and resources<br><br>Research-facing professional requirements and expectations	While interests and intrinsic motivations of clinicians are important drivers of research engagement, clinicians are most likely to become engaged in research if they are exposed to professional incentives, and have access to mentors, research training opportunities and adequate supports. Different research experience levels and approaches to learning are catered to by availability of different research training opportunities, often involving university collaboration.
Visible signals of research value and valued research types	Communication of research activity and achievements	Regular communication of research opportunities, activity and achievements across all research types and disciplines exposes clinicians to research and motivates engagement.
Characteristics of an individual's clinical role	Misaligned funding and reporting structures<br><br>Research-facing professional requirements and expectations<br><br>Research literacy at all levels<br><br>Workforce turnover and shortages	Funding and reporting structures that create disincentives for research, and high clinical workloads, were the main factors limiting clinicians' ability to engage in research. Expectations relating to research involvement are different for staff at different levels and disciplines, and workforce capacities and research literacy of managers also differ between locations, contributing to variable experiences of support and capacity between staff.
"Research culture" and multidisciplinary collaboration	Research literacy at all levels<br><br>Risk aversion to clinical practice changes	Cultures enabling research engagement and translation are multidisciplinary but also underpinned by staff understanding of research and its role within the health service. Low levels of understanding, combined with risk aversion to clinical practice change and high clinical workloads, are major contextual barriers.
Leaders' understanding and expectations relating to research	Research literacy at all levels	The ability of staff at all levels – not just leadership – to understand research is necessary for all types of impact to occur.
Responsiveness of research to patient and population health issues	Governance structures supporting multi-organisation collaboration<br><br>Clinicians' individual motivations and connections<br><br>Lack of strategic prioritisation of research<br><br>Few organisational structures to support translation<br><br>Research literacy at all levels	A wide range of contextual factors influence the ability of clinicians in the health service to translate research into practice, policy and workforce impacts. Responsiveness of research to real world issues and priorities is an enabler, but other factors including organisational structures to support research translation, underpinned by understanding of research and translation at all levels, and collaboration between different organisations are also necessary to enable research impacts beyond increasing research activity.
Reporting and funding models	Misaligned funding and reporting structures	This was identified as a major barrier to research impact in phases 1&2 of the study.
